# Factors associated with attrition, mortality, and loss to follow up after antiretroviral therapy initiation: data from an HIV cohort study in India

**DOI:** 10.3402/gha.v6i0.21682

**Published:** 2013-09-12

**Authors:** Gerardo Alvarez-Uria, Praveen K. Naik, Raghavakalyan Pakam, Manoranjan Midde

**Affiliations:** Department of Infectious Diseases, Rural Development Trust Hospital, Bathalapalli, India

**Keywords:** developing countries, rural health, highly active antiretroviral therapy, lost to follow up, attrition, mortality

## Abstract

**Background:**

Studies from sub-Saharan Africa have shown high incidence of attrition due to mortality or loss to follow-up (LTFU) after initiating antiretroviral therapy (ART). India is the third largest country in the world in terms of HIV infected people, but predictors of attrition after ART initiation are not well known.

**Design:**

We describe factors associated with attrition, mortality, and LTFU in 3,159 HIV infected patients who initiated ART between 1 January 2007 and 4 November 2011 in an HIV cohort study in India. The study included 6,852 person-years with a mean follow-up of 2.17 years.

**Results:**

After 5 years of follow-up, the estimated cumulative incidence of attrition was 37.7%. There was no significant difference between attrition due to mortality and attrition due to LTFU. Having CD4 counts <100 cells/µl and being homeless [adjusted hazard ratio (aHR) 3.1, 95% confidence interval (CI) 2.6–3.8] were associated with a higher risk of attrition, and female gender (aHR 0.64, 95% CI 0.6–0.8) was associated with a reduced risk of attrition. Living near a town (aHR 0.82, 95% CI 0.7–0.999) was associated with a reduced risk of mortality. Being single (aHR 1.6, 95% CI 1.2–2.3), illiteracy (aHR 1.3, 95% CI 1.1–1.6), and age <25 years (aHR 1.3, 95% CI 1–1.8) were associated with an increased risk of LTFU. Although the cumulative incidence of attrition in patients diagnosed with tuberculosis after ART initiation was 47.4%, patients who started anti-tuberculous treatment before ART had similar attrition to patients without tuberculosis (36 vs. 35.2%, *P*=0.19) after four years of follow-up.

**Conclusions:**

In this cohort study, the attrition was similar to the one found in sub-Saharan Africa. Earlier initiation of ART, improving the diagnosis of tuberculosis before initiating ART, and giving more support to those patients at higher risk of attrition could potentially reduce the mortality and LTFU after ART initiation.

By the end of 2011, 8 million people had started antiretroviral therapy (ART) worldwide (1). The roll out of ART has added 15 million life-years in low- and middle-income countries, and it is estimated that it can reduce the HIV incidence by 17–32% (1, 2).

With 2.1 million people living with HIV (3), India has the third largest burden of HIV worldwide. The HIV epidemic in India has been considered concentrated in high-risk groups (female sex workers, men who have sex with men, transgender, and injecting drug users) who transmit the virus to the general population through a bridge population (male migrants and truckers). Targeted interventions in these groups have achieved a dramatic 57% reduction in the incidence of HIV, according to governmental data (3). However, some epidemiological studies suggest a more generalised distribution of HIV in the population (4–7).

By December 2012, 1.7 million people living with HIV had been registered in Government ART centres, of whom 604,987 had started ART under the national programme (8). Studies from sub-Saharan Africa had shown that the cumulative incidence of attrition after 3 years of follow-up can be up to 35% (9). However research studies investigating predictors of mortality or loss to follow up (LTFU) after ART initiation are scarce in India (10). A better understanding of the risk factors associated with attrition in India could be helpful to design interventions to reduce mortality and LTFU in patients who initiate ART. The aim of this study is to describe the attrition after ART initiation in a large cohort of patients in Anantapur, India.

## Methods

### Setting

The study was performed in Anantapur, a district situated in the State of Andhra Pradesh, India. In Anantapur, 72% of the population live in rural areas (11), and there is >1% prevalence of HIV infection in antenatal clinics (12). The HIV epidemic in Anantapur is largely driven by heterosexual transmission, and it is characterised by poor socio-economic conditions and high levels of illiteracy (6). Rural Development Trust (RDT) is a non-governmental organisation that provides medical care to HIV infected people free of charge, including medicines, consultations, or hospital admission charges. In Bathalapalli RDT Hospital, CD4 count enumeration and ART are provided free of charge by the Indian Government under a public–private partnership. During the study period, ART was also provided by another ART centre in the district (Anantapur ART centre), and ART was initiated according to the Indian National Guidelines (13–15), so the CD4 cell count threshold for initiating ART was 250 cells/µl.

### Study design

The Vicente Ferrer HIV Cohort Study is an open cohort study of all HIV infected patients who have attended RDT hospitals since June 2006. Routine clinical data from patients were collected prospectively since September 2009, and retrospectively from June 2006 to September 2009. The characteristics of the cohort have been described in detail elsewhere (6).

### Study population

For this study, we selected HIV infected adults (>15 years) living in Anantapur who initiated ART between 1 January 2007 and 4 November 2011. Patients transferred to other ART centres after initiating ART were not included in the analysis. The selection of patients from the database was executed on 14 September 2012 (end of the study period).

Patients’ LTFU were routinely searched for by phone calls and home visits by a group of 31 outreach workers distributed throughout the district, and in those patients who had died, relatives were asked the date of death of the patients.

### Definitions

Designation of the community of patients was performed by self-identification. Scheduled caste community is marginalised in the traditional Hindu caste hierarchy and, therefore, suffers social and economic exclusion and disadvantage (16). Scheduled tribe community is generally geographically isolated with limited economic and social contact with the rest of the population (16). Scheduled castes and scheduled tribes are considered socially disadvantaged communities and are supported by positive discrimination schemes operated by the Government of India (17). Patients were considered as living near an ART centre if they lived in a mandal (administrative subdivision of districts in Andhra Pradesh; e.g. Anantapur district has 64 mandals) with an ART centre, or lived next to a mandal with an ART centre. Patients were considered as living near a town when they lived in a mandal containing a town with a population >100,000 people. Towns have better communications than rural areas. Poverty was defined as living with less than 1,000 Indian rupees per month (approximately US$18 as of May 2013). Illiteracy was defined as not being able to read or write.

### Ethics statement

The study was approved by the Ethical Committee of the Rural Development Trust Hospital. Written informed consent was given by patients or caretakers for their information to be stored in the study database and used for research.

### Statistical analysis

Statistical analysis was performed using Stata Statistical Software (Stata Corporation. Release 11. College Station, TX, USA). To investigate predictors of attrition, time-to-event methods were used. Time was measured from ART initiation to death. Patients who did not die during the study period were censored at their last visit to the clinics. Patients who did not come to the clinics for at least 180 days after their last scheduled appointment were considered LTFU (18). Attrition, mortality, and LTFU rates were calculated by summing the number of patients who experienced the event (attrition, death, or LTFU) during a particular period of time divided by the total number of years of follow-up during this period. Multivariable analysis was performed by Cox regression proportional hazard models. The proportional hazard assumption was assessed performing log–log survival curves based on Schoenfeld residuals (19). Missing values were imputed using multiple imputations by chained equation assuming missing at random (20). The variables that were imputed were poverty (33 missing values), homelessness (46 missing values), illiteracy (4 missing values), and marital status (6 missing values). We used Kaplan–Meier survival estimates to calculate cumulative incidences of attrition. Cumulative incidences of mortality and LTFU were calculated using competing risk analysis (stcompet command in Stata) (21).

## Results

We identified 3,159 patients who initiated ART. Baseline characteristics of the patients and rates of attrition, mortality, and LTFU are described in Table 1. Forty-one percent of patients were women, two thirds were married, and the median age was 33.8 years [interquartile range (IQR) 28–40). One fourth belonged to socially disadvantaged communities, more than half were illiterate, 7% were homeless, and more than one third had a monthly income <1,000 Indian rupees. One third of patients were living near an ART centre, and 44% were living near a town. The median CD4 cell count was 140 cells/µl (IQR 84–195). Thirteen percent were diagnosed with tuberculosis within 3 months before ART initiation, and 7.5% within 3 months after ART initiation.

**Table 1:** Baseline characteristics and rates of attrition, mortality, and loss to follow up of patients initiating antiretroviral therapy

	Baseline characteristics *N* (%) (*n=*3,159)	Attrition rate/100 py (overall=14.1)	Mortality rate/100 py (overall=7)	Loss to follow up rate/100 py (overall=7.1)
Female gender	1,283 (40.61)	10.4	4.9	5.4
Age (years)				
< 25	448 (14.18)	13.5	5.1	8.4
25–35	1,426 (45.14)	13.1	6.5	6.6
35–45	882 (27.92)	14.5	7.7	6.7
> 45	403 (12.76)	18.1	9.3	8.8
Disadvantaged community	796 (25.2)	15.7	7.8	7.9
Illiteracy	1,800 (57.05)	14.7	7.2	7.5
Marital status				
Divorced/separated	185 (5.87)	14.4	7.3	7.1
Married	2,200 (69.77)	14.5	7.3	7.2
Single	172 (5.46)	22.8	9.4	13.4
Widowed	596 (18.9)	10.8	5.2	5.5
Homeless	213 (6.84)	40	19.8	20.1
Living near an ART centre	1,042 (32.99)	12.8	6.9	5.9
Living near a town	1,400 (44.32)	13.3	6.1	7.2
Poverty	1,142 (36.53)	11.9	5.7	6.2
CD4 before ART initiation				
< 50	375 (11.87)	24.2	13.6	10.7
50–100	641 (20.29)	17.9	10.1	7.8
100–150	700 (22.16)	13.3	6.5	6.8
150–200	738 (23.36)	10.8	5	5.9
> 200	705 (22.32)	10.7	3.9	6.7
TB diagnosis				
< 3 months before ART	388 (12.3)	16.5	10.7	5.8
< 3 months after ART	237 (7.5)	28.4	19.2	9.2
No TB	2,534 (82.2)	12.9	5.7	7.2
Year of ART eligibility				
2007	482 (15.26)	11.2	4.1	7.1
2008	556 (17.6)	14.2	6.7	7.6
2009	697 (22.06)	12.4	5.6	6.8
2010	809 (25.61)	16.6	9	7.6
2011	615 (19.47)	21.2	15	6.2

ART, antiretroviral therapy; TB, tuberculosis; py, person-years.

**Fig. 1:**
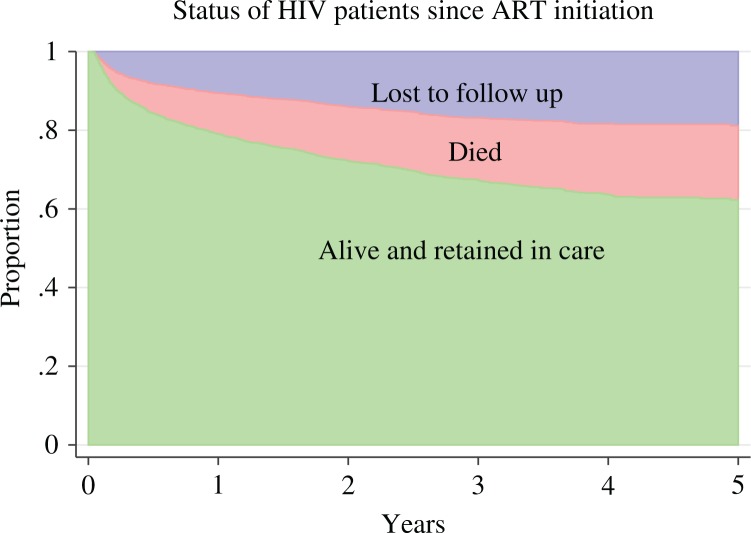
Stacked graph of the status of HIV patients since antiretroviral therapy (ART) initiation.

The study included 6,852 person-years (PY) with a mean follow-up of 2.17 years (standard deviation 1.5); 478 (15.1%) patients died, and 489 (15.5%) were LTFU. The overall attrition rate was 14.1 per 100 PY, the mortality rate was 7 per 100 PY, and the LTFU rate was 7.1 per 100 PY. The median time to death was 0.52 years (IQR, 0.18–1.3), and the median time to be LTFU was 0.45 years (0.16–1.38). Figure 1 presents a stacked graph of the status of HIV patients since ART initiation. The attrition rate after ART initiation was 34.8 per 100 PY during the first semester (0–6 months), 12.9 per 100 PY during the second semester (6–12 months), 9.1 per 100 PY during the second year (12–24 months), 6.9 per 100 PY during the third year (24–36 months), 5.8 per 100 PY during the fourth year (36–48 months), and 2.4 per 100 PY during the fifth year (48–60 months). The cumulative incidence of attrition, mortality, and LTFU after 6 months, 1 year, 2 years, 3 years, 4 years, and 5 years of follow-up is presented in Table 2. The estimated cumulative incidence of attrition for any reason was 37.7% [95% confidence interval (CI) 35.5–40] at 5 years.

**Table 2:** Cumulative incidence and 95% confidence interval of attrition (mortality or loss to follow up), mortality, and loss to follow up at 0.5, 1, 2, 3, 4, and 5 years after antiretroviral therapy initiation

	Cumulative incidence (95% CI)		

	Attrition	Mortality	Loss to follow up
6 months	15.8% (14.5–17.1)	7.5% (6.6–8.5)	8.2% (7.3–9.2)
1 year	21% (19.6–22.5)	10.4% (9.4–11.5)	10.6% (9.5–11.7)
2 years	27.9% (26.3–29.5)	13.8% (12.6–15.1)	14.1% (12.9–15.4)
3 years	32.7% (30.9–34.5)	15.8% (14.5–17.2)	16.8% (15.4–18.3)
4 years	36.4% (34.3–38.5)	18% (16.4–19.7)	18.4% (16.8–20)
5 years	37.7% (35.5–40.1)	18.9% (17.1–20.7)	18.9% (17.1–20.6)

CI, confidence interval.

**Fig. 2:**
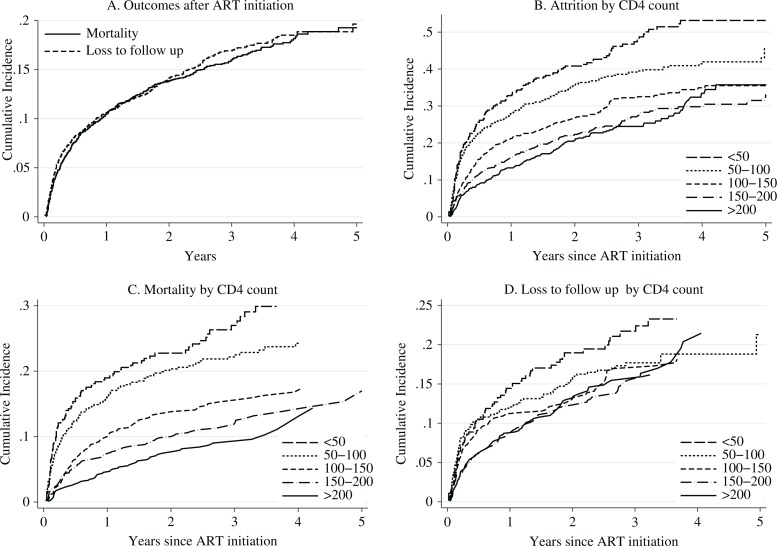
Cumulative incidence of (A) mortality and loss to follow up, (B) attrition by CD4 lymphocyte count, (C) mortality by CD4 lymphocyte count, and (D) loss to follow up by CD4 lymphocyte count.

Figure 2 presents the cumulative incidence of attrition, mortality, and LTFU stratified by CD4 cell counts. The cumulative incidence curve of mortality was not statistically different from the cumulative incidence curve of LTFU (log–rank test *P* =0.65) (Fig. 2A). The mortality rate after ART initiation was 16.7 per 100 PY during the first semester, 7 per 100 PY during the second semester, 4.5 per 100 PY during the second year, 3 per 100 PY during the third year, 3.4 per 100 PY during the fourth year, and 1.7 per 100 PY during the fifth year. The LTFU rate after ART initiation was 18.1 per 100 PY during the first semester, 5.9 per 100 PY during the second semester, 4.6 per 100 PY during the second year, 4 per 100 PY during the third year, 2.4 per 100 PY during the fourth year, and 0.7 per 100 PY during the fifth year.

The cumulative incidence curves of attrition by CD4 cell counts showed three patterns of attrition: patients with CD4 counts <50 cells/µl, those with CD4 counts 50–100 cells/µl, and those with CD4 counts >100 cells/µl (Fig. 2B). Mortality was inversely proportional to the CD4 cell counts of the patients (Fig. 2C). Cumulative incidence curves of LTFU showed three patterns of attrition: patients with CD4 counts <50 cells/µl, those with CD4 counts 50–150 cells/µl, and those with CD4 counts >150 cells/µl (Fig. 2D). However, patients with CD4 counts >50 cells/µl had similar proportions of LTFU after 3 years of follow-up.

**Fig. 3:**
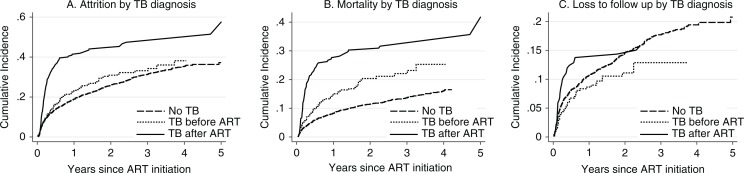
(A) Attrition, (B) mortality, and (C) loss to follow up by the timing of tuberculosis diagnosis. ART, antiretroviral therapy; TB, tuberculosis.

Figure 3 presents the cumulative incidence of attrition, mortality, and LTFU by the timing of tuberculosis diagnosis. Patients diagnosed with tuberculosis after ART initiation had higher incidence of attrition (47.4%) and mortality (31.6%) after 4 years of follow-up. This group also had a higher incidence of LTFU during the first 2 years of follow-up (Fig. 3C). Those patients diagnosed with tuberculosis before ART initiation had a similar incidence of attrition (36 vs. 35.2%, log–rank test *P*=0.19), higher incidence of mortality (23.2 vs. 16%, log–rank test *P*<0.001), and lower incidence of LTFU (12.9 vs. 19.3%, log–rank test *P*=0.045) than patients without tuberculosis after 4 years of follow-up.

**Fig. 4:**
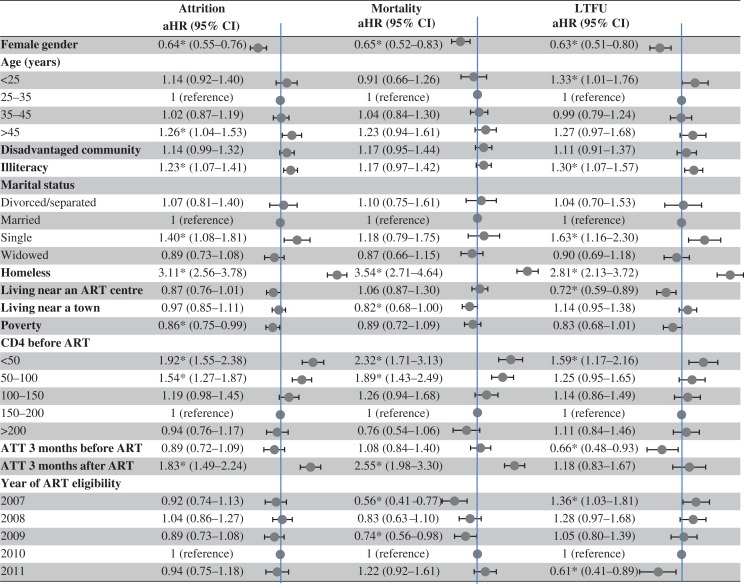
Cox regression analysis of factors associated with attrition (mortality or loss to follow up), mortality, and loss to follow up. *P value <0.05. aHR, adjusted hazard ratio by Cox regression analysis; ART, antiretroviral therapy; ATT, anti-tuberculous treatment; CI, confidence interval; LTFU, loss to follow up.

Figure 4 presents the Cox regression analysis of factors associated with attrition, mortality, and LTFU. Factors associated with attrition were being homeless [adjusted hazard ratio (aHR) 3.11, 95% CI 2.56–3.78); CD4 count <50 cells/µl (aHR 1.92, 95% CI 1.55–2.38); CD4 count 50–100 cells/µl (aHR 1.54, 95% CI 1.27–1.87); initiating anti-tuberculous treatment within 3 months after ART initiation (aHR 1.83, 95% CI 1.49–2.24); being single, age >45 years (aHR 1.26, 95% CI 1.04–1.53); and illiteracy (aHR 1.23, 95% CI 1.07–1.41). Female gender (aHR 0.64, 95% CI 0.55–0.76) and poverty (aHR 0.86, 95% CI 0.75–0.99) were factors associated with a lower risk of attrition. Factors associated with mortality were being homeless (aHR 3.54, 95% CI 2.71–4.64); CD4 count <50 cells/µl (aHR 2.32, 95% CI 1.71–3.13); CD4 count 50–100 cells/µl (aHR 1.89, 95% CI 1.43–2.49); and initiating anti-tuberculosis treatment within 3 months after ART initiation (aHR 2.55, 95% CI 1.98–3.3). Female gender (aHR 0.65, 95% CI 0.52–0.83) and living near a town (aHR 0.82, 95% CI 0.68–1) were factors associated with a lower risk of mortality. Factors associated with LTFU were being homeless (aHR 2.81, 95% CI 2.13–3.72); being single (aHR 1.63, 95% CI 1.16–2.3); CD4 count <50 cells/µl (aHR 1.59, 95% CI 1.17–2.16); age <25 years (aHR 1.33, 95% CI 1.01–1.76); and illiteracy (aHR 1.3, 95% CI 1.07–1.57). Female gender (aHR 0.63, 95% CI 0.51–0.8); initiating anti-tuberculous treatment within 3 months before ART initiation (aHR 0.66, 95% CI 0.48–0.93); and living near an ART centre (aHR 0.72, 95% CI 0.59–0.89) were factors associated with a lower risk of LTFU. Although an earlier calendar year of ART initiation was not associated with attrition after ART initiation, it was associated with an increased risk of LTFU and a reduced risk of death, suggesting that it was more difficult to document the mortality at earlier calendar years.

## Discussion

In this study, it was demonstrated that nearly 40% of patients who initiate ART die or are LTFU after 5 years of follow-up. This staggering figure is in line with other studies from sub-Saharan Africa (9), and with a study performed in an urban setting in North India (10), which found that 35 and 39% of patients had died or were LTFU after 3 years of follow-up, respectively. However, the attrition was higher than the ones reported in recent studies from China (24%), Thailand (19%), and South Africa (21–26%) (22–24). These findings highlight the urgent need to reduce the attrition after ART initiation of HIV infected patients in India.

Patients with CD4 counts <100 cells/µl were at higher risk of attrition. Starting ART at higher CD4 cell counts could have a major impact on reducing the mortality and LTFU after ART initiation. Current Indian guidelines have raised the CD4 cell count threshold for initiation of ART to 350 cells/µl (25), and this threshold has been recently increased to 500 cells/µl by the World Health Organization (26). However, in our setting, two thirds of patients are diagnosed with HIV when their CD4 counts are <350 cells/µl (27). Increasing the CD4 cell count threshold for starting ART will not increase the CD4 cell count of patients at ART initiation unless this is accompanied by interventions to achieve earlier diagnosis of HIV and to improve the linkage between HIV-diagnosis and ART centres (27, 28).

In this study, we describe socio-demographic factors associated with attrition in India, which can be used to design interventions aimed at giving extra support to groups of patients having a higher risk of attrition. Male gender, illiteracy, being homeless, and being >45 years old were factors associated with an increased risk of attrition. Patients who were <25 years old or single were more likely to be LTFU. Typically, HIV infected individuals who are found to be eligible for ART receive two or three sessions of ART preparedness counselling before initiating ART (25). HIV programmes in India should take into consideration that these groups of patients at higher risk of attrition may need a more intensive counselling before initiating ART (24).

In line with two South African studies (22, 29), attrition was more common during the first year of follow-up. Studies from resource-limited settings have shown that the most common reasons given by patients to not come back to the clinics are financial and fear of harsh treatment by healthcare workers (30, 31). Implementing strategies to provide social and economic support during the first year of ART could reduce the programme attrition because patients who were retained in care for 1 year were less likely to be LTFU (32). Moreover, campaigns to educate healthcare providers about the benefits of using positive rather than negative reinforcement in patients who miss appointments to the clinics, and to reduce the stigma and discrimination in healthcare facilities could be beneficial (31).

In accordance with a South African study (22), factors associated with mortality were similar to factors associated with LTFU. However, differences between predictors of mortality and LTFU were also found. Being single or <25 years old was associated with an increased risk of LTFU, but not with an increased risk of mortality. People living far from a town had a higher risk of mortality, but did not have a higher risk of LTFU. The higher risk of death of people living in rural areas is probably related to poorer access to medical care in rural areas of India (33). Patients living near an ART centre were less likely to be LTFU, which supports the current policy of decentralisation of ART centres of the Indian Government (34).

Patients who initiated anti-tuberculous treatment after ART initiation had an increased risk of attrition. However, initiating anti-tuberculous treatment before ART was not associated with an increased risk of attrition and was associated with a reduced risk of LTFU in the multivariable analysis. The increased risk of death observed when initiating ART before anti-tuberculous treatment confirms previous findings from our cohort and other resource-limited settings (35, 36). Although these results suggest that HIV patients living in areas with a high prevalence of tuberculosis should be thoroughly investigated for tuberculosis before ART initiation, current Indian guidelines do not have a protocol to rule out tuberculosis before starting ART (25).

The study had some limitations. Patients LTFU may not be lost forever because they may reengage in the future or enrol in other ART centres. However, in a systematic review and meta-analysis of the mortality of patients LTFU in sub-Saharan Africa, it was estimated that the mortality of patients LTFU can be nearly 50% in programmes with a 20% rate of LTFU (30). In a community tracking survey of patients LTFU in Ethiopia (37), nearly half of patients LTFU died, most deaths occurred during the first 6 months of loss, and the mortality was particularly higher in those patients with lower CD4 cell counts or tuberculosis. The high mortality of patients LTFU may explain the similarities between the predictors of mortality and LTFU found in our study. Those variables related to LTFU, but not to mortality (i.e. younger age, being single, illiteracy, and not living near an ART centre), may be useful to identify patients with a lower risk of death among those LTFU.

## Conclusions

The results of this cohort study indicate that the attrition after ART initiation is extremely high. Nearly 40% of patients die or are LTFU. Having low CD4 cell counts, being homeless, and male gender were found to be factors strongly associated with attrition. Although the mortality of patients diagnosed with tuberculosis after ART initiation was very high, patients who started anti-tuberculosis treatment before ART had similar attrition to patients without tuberculosis. The results of this study indicate that intervention aimed at increasing the CD4 cell count of patients at the time of ART initiation, improving the diagnosis of tuberculosis before starting ART, and giving extra support to the groups of patients at higher risk of attrition could potentially reduce the mortality and LTFU of HIV programmes in India.
